# Reliability and Validity Study of Clinical Ultrasound Imaging on Lateral Curvature of Adolescent Idiopathic Scoliosis

**DOI:** 10.1371/journal.pone.0135264

**Published:** 2015-08-12

**Authors:** Q. Wang, M. Li, Edmond H. M. Lou, M. S. Wong

**Affiliations:** 1 Interdisciplinary Division of Biomedical Engineering, The Hong Kong Polytechnic University, Hong Kong, China; 2 Center of Rehabilitation Medicine, West China Hospital, Sichuan University, Chengdu, China; 3 Institute for Disaster Management and Reconstruction, Sichuan University-The Hong Kong Polytechnic University, Chengdu, China; 4 Department of Surgery, Glenrose Rehabilitation Research Centre, University of Alberta, Edmonton, Canada; Van Andel Institute, UNITED STATES

## Abstract

**Background:**

Non-ionizing radiation imaging assessment has been advocated for the patients with adolescent idiopathic scoliosis (AIS). As one of the radiation-free methods, ultrasound imaging has gained growing attention in scoliosis assessment over the past decade. The center of laminae (COL) method has been proposed to measure the spinal curvature in the coronal plane of ultrasound image. However, the reliability and validity of this ultrasound method have not been validated in the clinical setting.

**Objectives:**

To evaluate the reliability and validity of clinical ultrasound imaging on lateral curvature measurements of AIS with their corresponding magnetic resonance imaging (MRI) measurements.

**Methods:**

Thirty curves (ranged 10.2°–68.2°) from sixteen patients with AIS were eligible for this study. The ultrasound scan was performed using a 3-D ultrasound unit within the same morning of MRI examination. Two researchers were involved in data collection of these two examinations. The COL method was used to measure the coronal curvature in ultrasound image, compared with the Cobb method in MRI. The intra- and inter-rater reliability of the COL method was evaluated by intra-class correlation coefficient (ICC). The validity of this method was analyzed by paired Student’s *t*-test, Bland–Altman statistics and Pearson correlation coefficient. The level of significance was set as 0.05.

**Results:**

The COL method showed high intra- and inter-rater reliabilities (both with ICC (2, K) >0.9, p<0.05) to measure the coronal curvature. Compared with Cobb method, COL method showed no significant difference (p<0.05) when measuring coronal curvature. Furthermore, Bland-Altman method demonstrated an agreement between these two methods, and Pearson’s correlation coefficient (r) was high (r>0.9, p<0.05).

**Conclusion:**

The ultrasound imaging could provide a reliable and valid measurement of spinal curvature in the coronal plane using the COL method. Further research is needed to validate the proposed ultrasound measurement in larger clinical trial and to optimize the ultrasound scanning and measuring procedure.

## Introduction

Adolescent idiopathic scoliosis (AIS) is a three-dimensional spinal deformity characterized by lateral curvature and vertebral rotation of spine. It occurs in approximately 3% of adolescents with unknown reasons [1, 2]. Nowadays, the radiographic assessment of scoliotic spine continues to be the most widely used method in a scoliosis clinic. In standing posterior-anterior radiographs, the spinal curvature can be assessed with the Cobb method, which was adopted by the Scoliosis Research Society (SRS) as the standard reference method to diagnose and monitor AIS [3]. The intra- and inter-rater variation of the Cobb method has been reported to be within the range of 1.2° to 7° [4–6], and the intra-class correlation coefficient values 0.88 to 0.99 [6–8].

In routine clinical practice, radiographic assessments are performed throughout the course of treatment of the patients with AIS. However, the frequency of radiation exposure in monitoring scoliosis concerns many adolescents and their parents in light of evidence that cumulative radiation exposure could increase cancer risk [9]. In addition, radiographic assessment of scoliotic spine is limited in the coronal and sagittal planes, which represent a simplification of the true 3-dimensional (3-D) spinal deformity involved in scoliosis. Thus, attempts to reduce radiation exposure in adolescents and visualization of 3-D characteristics of scoliotic spine have led researchers to develop new imaging technologies, such as ultrasound imaging, stereo-radiography (EOS), surface topography and magnetic resonance imaging (MRI).

Among various imaging technologies, ultrasound imaging has some superior characteristics such as radiation-free, cost effective and easy to operate. With the advent of 3-D reconstruction technique, ultrasound imaging has been developed to quantify the 3-D nature of scoliotic spine [10–12]. Thus, the use of ultrasound in scoliosis assessment has gained considerable attention over the past decade. A series of the related research have been conducted in Canada [13–16], Hong Kong [11, 17–19], Japan [20], Australia [21], Netherlands [10] and other places.

The landmarks such as spinous processes [11, 16], transverse processes [22] and laminae [13, 15] have been identified in ultrasound images. The feasibility of using these landmarks to assess the spinal curvature has been studied. In 1988, the first attempt to use ultrasound to assess spinal curvature was made by Letts et al., who applied ultrasonic digitization to identify spinous process and document spinal curvature using Ferguson method [16]. In recent years, Wong et al. used ultrasound to estimate the Cobb angle through spinous process angle (SPA) method [11], by which the optimal location of pressure pad of spinal orthosis could be determined during the fitting procedure of orthosis [11, 18]. Additionally, Ungi et al. showed that the transverse process angle (TRA) obtained from ultrasound could be correlated with the Cobb angle when measuring coronal curvature [22]. At the same time, in a phantom study the center of laminae (COL) method in ultrasound has been proposed by Lou and his colleague to approximate the Cobb angle in the coronal plane. The intra- and inter-reliability of the COL method was showed to be high for the patients with AIS ranged from 12° to 45°. Furthermore, the correlation was found between the COL method in ultrasound and the Cobb method in radiograph [23, 24]; the measurement difference between these two methods was less than 5° [24].

The above studies supported the feasibility of using ultrasound in the assessment of spinal curvature for the patients with AIS. However, most of the relevant results currently available regarding ultrasound assessment of spinal curvature were derived in phantom studies, but not in clinical trials. For this reason, it is warranted to systematically validate the proposed ultrasound measurement of spinal curvature in a clinical setting. Thus, the purpose of this study was to evaluate the reliability and validity of clinical ultrasound imaging on lateral curvature measurement of AIS, in comparison with the corresponding magnetic resonance imaging (MRI) measurement. Since the COL method in ultrasound has been proposed both in the phantom and pilot studies, the results of this study would provide a comprehensive clinical evidence to further validate the COL method in ultrasound assessment of the patients with AIS.

## Materials and Methods

### Clinical subjects

The subject selection criteria were as follows: 1) female adolescents; 2) age: 10–18 years; 3) Cobb angle: 10°-80°; 4) no prior surgical treatment; 5) out-of-brace MRI examination of the whole spine on the study day.

Sixteen female subjects with AIS (aged 15.4 ± 2.6 years) were recruited from the local scoliosis clinic. Human ethical approval was granted from both the Human Subjects Ethics Sub-committee of the Hong Kong Polytechnic University and the Joint Chinese University of Hong Kong-New Territories East Cluster Clinical Research Ethics Committee. All subjects signed written consent prior to the study.

### Data acquisition

**Fig 1:**
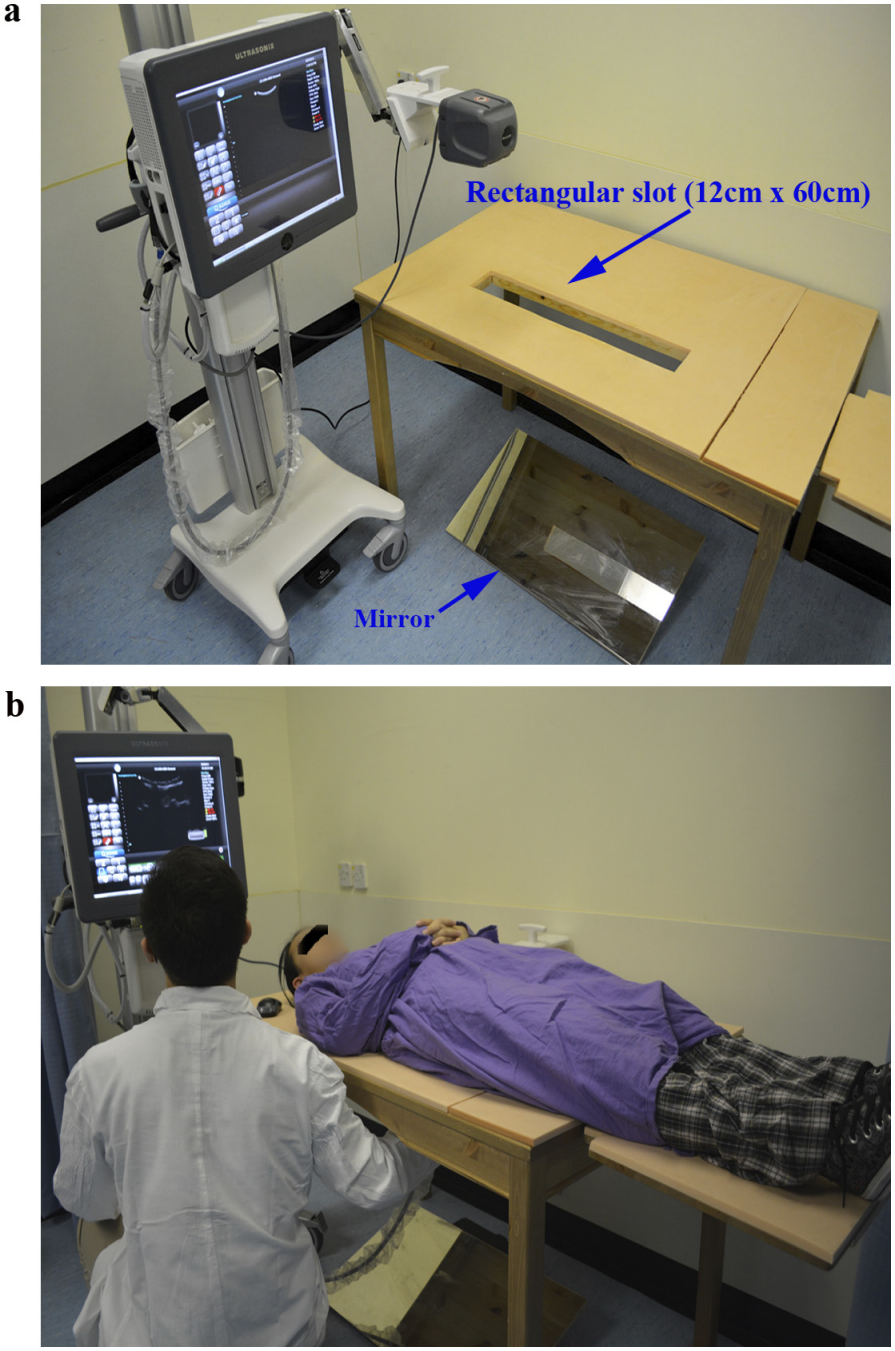
Clinical ultrasound system and ultrasound scan. (a) 3-D Ultrasound unit and a purpose-design couch; (b) Ultrasound scanning at supine position.

To minimize the potential errors, ultrasound and MRI scans of the full spine were arranged on the same morning (generally within 3 hours). The ultrasound scan was performed using a 3-D SonixTABLET ultrasound unit coupled with the SonixGPS and a C5-2/60 Convex transducer (Ultrasonix, Canada) (Fig 1a). The parameters of ultrasound scan were set as follows: frequency 2.5 MHz, penetration depth 18cm, gain 10%. A purpose-design couch with a central rectangular slot (size: 12 cm [width] x 60 cm [length]) was used to facilitate ultrasound scanning at supine position, which was similar to the position of MRI scanning in routine clinical examination (Fig 1a). In addtion, a mirror was placed under the slot in order to assist the raters to correctly move the ultrasound transducer along the spine. The subjects wore a gown with the back opened (about 8 cm). The spinous processes from C7 to S1 were palpated and marked on the subjects' back by a water soluble marker. Ultrasound gel was applied to ensure a good contact between the transducer and the subject’s back. Ultrasound scanning was performed continuously along the coronal curvature from C7 to S1, with the subjects lying on the scanning couch (Fig 1b). Each subject underwent 6 scans (2 raters and each with 3 scans) and it took less than a minute per scan.

To compare with the ultrasound measurements, MRI data were collected using a 3.0T MR scanner and a spine array coil (Achieva, Philips Medical Systems, Netherlands). Before MRI scanning, a level meter was used to test the relative position of the anterior superior iliac spines (ASIS) at both side of pelvis. Then the soft cushions or pads would be applied to adjust the subject's pelvis to ensure the both ASIS to be in horizontal line during MRI scanning. The MRI data were exported from the picture archiving and communication system (PACS).

### Data measurement

**Fig 2:**
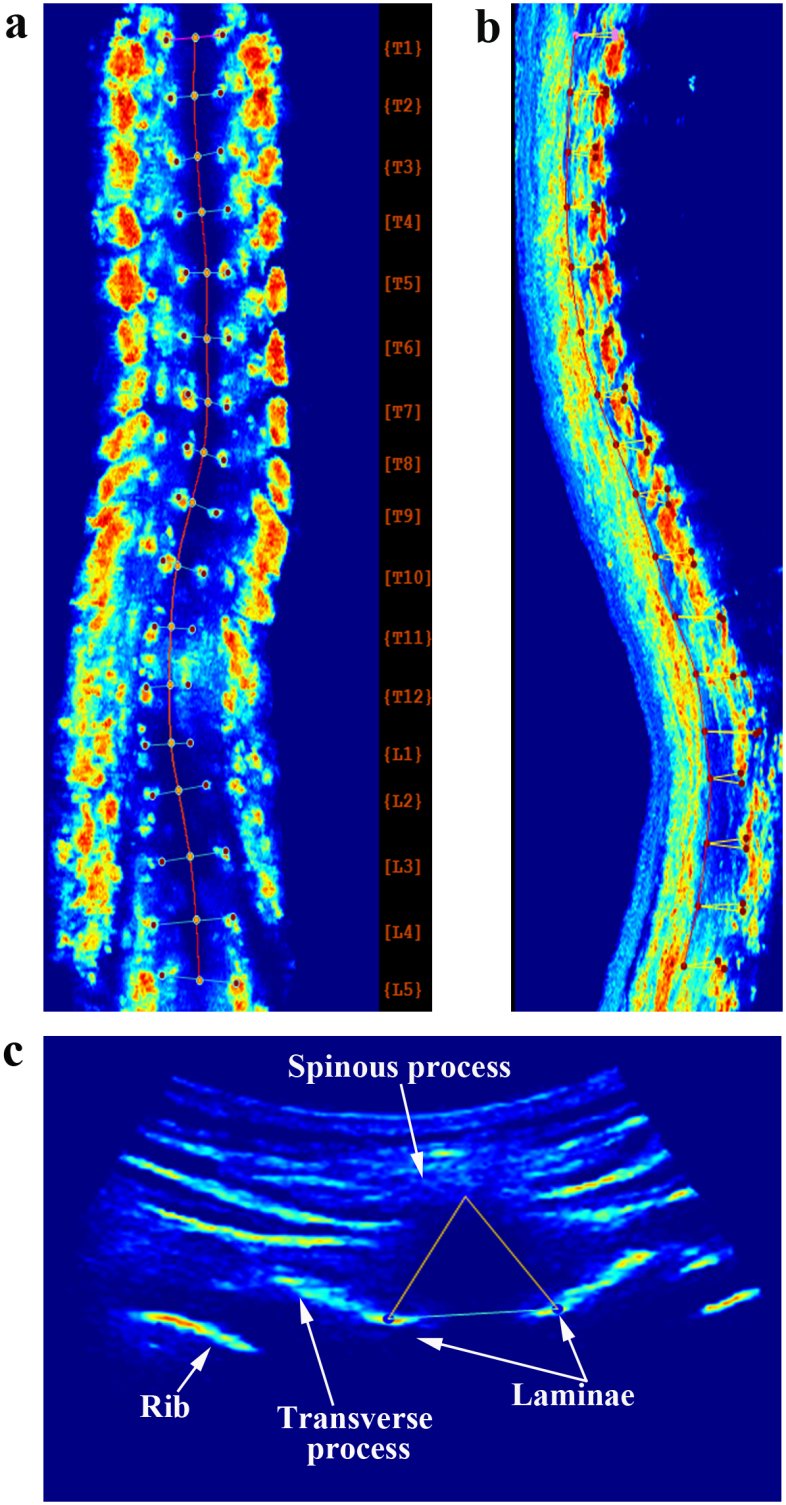
3-D reconstructed ultrasound images of scoliotic spine. (a) Coronal plane; (b) Sagittal plane; (c) Transverse plane.

**Fig 3:**
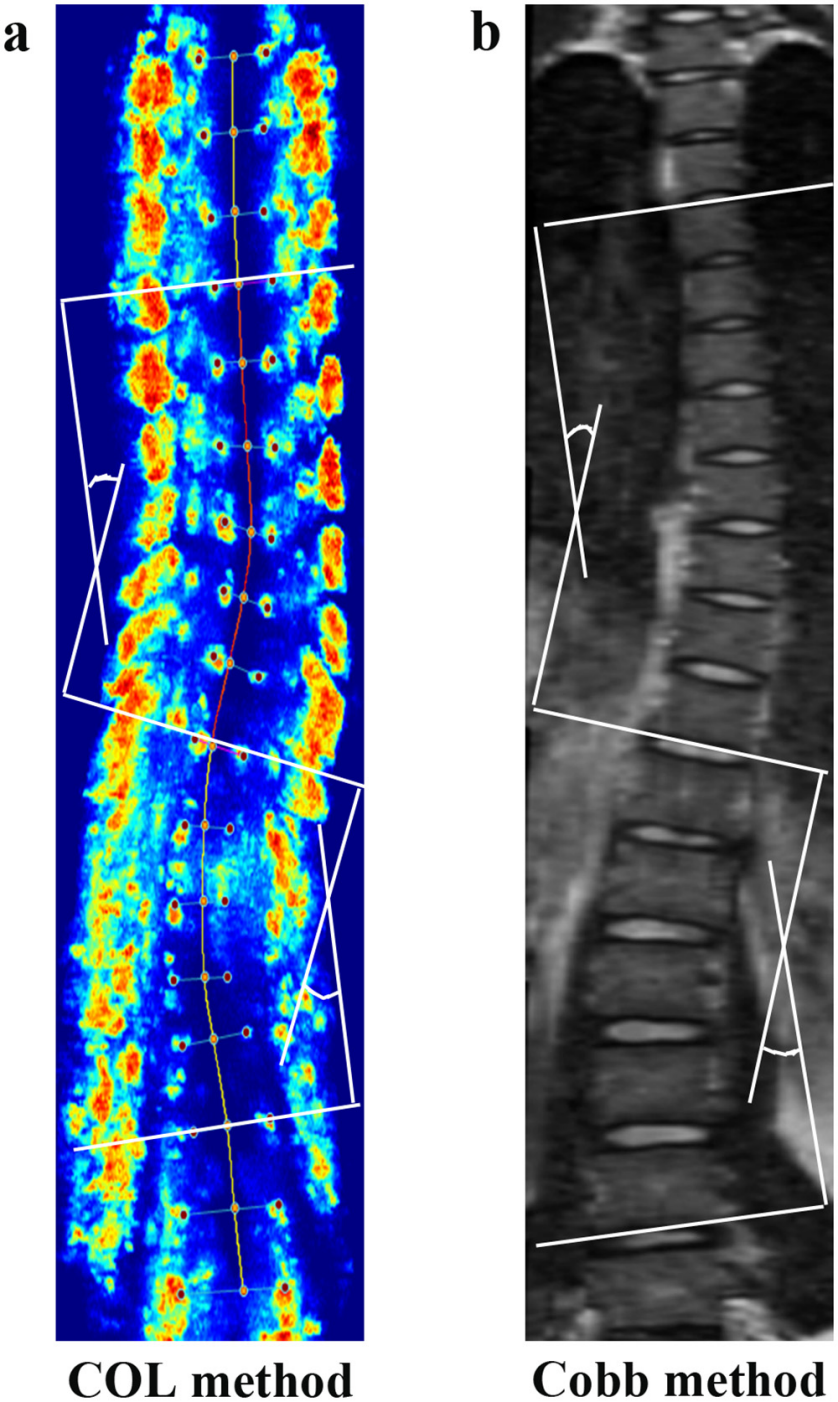
Spinal curvature measurement using. (a) Center of laminae (COL) method in ultrasound; (b) Cobb method in MRI.

For ultrasound assessments, the reconstructed 3-D ultrasound images of vertebra were shown in the 3 orthogonal planes (coronal, sagittal and transverse) (Fig 2). The center of laminar (COL) method was applied to measure the spinal curvature angle in the coronal plane [15, 23, 24]. The laminae at each vertebral level were identified manually in the coronal plane of ultrasound image. The corresponding transverse plane was shown to aid in the adjustment of the location of the centers of the selected laminae. Then the lines were automatically drawn to join the centers of laminae at each level by the custom-developed software. The most titled lines above and below each curve would be selected manually by the two researchers as the levels of the upper and lower end-vertebrae. Similar to the Cobb angle measurement, the angle of the COL method was then calculated based on these two most titled lines by the software (Fig 3a).

For MRI measurements, the MRI images were exported into JPG format and processed by the Photoshop (Adobe Photoshop CS3 version, Adobe, USA) software. The apical, upper-& lower-end vertebrae were pre-defined in the coronal plane of MRI images. The Cobb method was used to measure the spinal curvature in the coronal plane (Fig 3b).

All ultrasound and MRI images were randomly assigned and measured 3 times by the two researchers at a minimum of 1 week interval. The two researchers had approximately 5 years and 2 years of experience respectively in ultrasound measurement of scoliotic spine. Prior to the study, each researcher was still required to practice ultrasound scanning at the supine position, data acquisition and measurement for more than 10 subjects. During the coronal curvature measurements they were blinded to all subjects' information and performed the measurements independently. The time required was approximately 5 minutes for the ultrasound data measurement.

### Statistical analysis

Statistical analyses were performed using the IBM SPSS Statistics Version 21 (IBM, USA). A *p*-value less than 0.05 were considered to be statistically significant. Statistical graphs were made with GraphPad Prism Version 6.01 software (GraphPad, La Jolla, California, USA). To assess the intra- and inter-rater reliability of ultrasound measurement, the intra-class correlation coefficient (ICC, [2, k]) with 95% confidence intervals (CI) was calculated. The Currier criteria for evaluating ICC values were adopted [25]: very reliable (0.80–1.0), moderately reliable (0.60–0.79), and questioned reliable (<0.60). In addition, the intra- and inter-rater measurement variation of the COL method and the Cobb method were evaluated using the mean absolute difference (MAD), standard deviations (SD) and standard error of measurement (SEM).

To evaluate the validity of ultrasound assessment, the paired Student’s *t*-test was used to compare the coronal curvatures obtained from the ultrasound and MRI assessments; the Bland–Altman method was used to examine the agreement between these two assessments [26, 27]; furthermore, the Pearson correlation analysis was applied to evaluate the correlation between ultrasound and MRI assessments.

## Results

Of the 16 AIS subjects, 3 had a single thoracic curve, 1 a single lumbar curve, 10 a double curve and 2 a triple curve, producing a total of 30 curves eligible for analysis in this study. The Cobb angles of these curves ranged from 10.2° to 68.2° and the average value was 21.7° ± 15.9°.

### Reliability of ultrasound assessment of spinal curvature in the coronal plane

The intra-rater variation and reliability for both ultrasound and MRI assessments of spinal curvature in the coronal plane were shown in Table 1. The MAD of the COL method in ultrasound ranged from 0.1° to 2.1°; the SD from 0.2° to 2.7°; and the SEM from 0.1° to 2.2°. All of these values were about 1° larger than those of the Cobb method in MRI, but were still characterized as the small variation between successive measurements. In addition, the ICC (2,k) values of the ultrasound measurements from both raters were above 0.9, indicating high intra-rater reliability.

**Table 1:** Intra-rater variation and reliability of coronal curvature assessments using ultrasound compared with MRI.

Methods	Raters	Curves, n	MAD^a^ (°)	SD^b^ (°)	SEM^c^ (°)	ICC [2,k] (95%CI) ^d^
Ultrasound (COL method)	R1	30	0.6 (0.1–1.3)	0.8 (0.2–1.7)	0.6 (0.2–1.4)	0.997 (0.994–0.998)
	R2	30	0.7 (0.1–2.1)	1.0 (0.2–2.7)	0.8 (0.1–2.2)	0.993 (0.986–0.996)
MRI (Cobb method)	R1	30	0.5 (0.2–1.2)	0.7 (0.2–1.6)	0.5 (0.2–1.3)	0.998 (0.996–0.999)
	R2	30	0.6 (0.1–1.1)	0.8 (0.2–1.5)	0.7 (0.1–1.2)	0.997 (0.994–0.998)

MAD^a^: mean absolute difference; SD^b^: standard deviation; SEM^c^: standard error of measurement; ICC^d^: intra-class correlation coefficient; CI^d^: confidence intervals.

The inter-rater variation and reliability of both assessments were shown in Table 2. Compared with MRI measurements, the ultrasound assessments showed similar MAD, SD and SEM between two raters. The ranges of MAD, SD and SEM were 0.4°-1.4°, 0.5°-2.2° and 0.5°-2.0° for the COL method in ultrasound, while 0.3°-1.8°, 0.4°-2.2° and 0.3°-2.0° for the Cobb method in MRI. The inter-rater ICC (2, k) values for both ultrasound and MRI measurements were greater than 0.9, which demonstrated high inter-reliability.

**Table 2:** Inter-rater variation and reliability of coronal curvature assessments using ultrasound compared with MRI.

Methods	Raters	Curves, n	MAD^a^ (°)	SD^b^ (°)	SEM^c^ (°)	ICC[2,k] (95%CI)^d^
Ultrasound (COL method)	R1 *vs*. R2	30	0.9(0.4–1.4)	1.1 (0.5–2.2)	1.0 (0.5–2.0)	0.995 (0.989–0.998)
MRI (Cobb method)	R1 *vs*. R2	30	0.8 (0.3–1.8)	1.1 (0.4–2.2)	1.0 (0.3–2.0)	0.995 (0.989–0.998)

MAD^a^: mean absolute difference; SD^b^: standard deviation; SEM^c^: standard error of measurement; ICC^d^: intra-class correlation coefficient; CI^d^: confidence intervals

These results suggested that the COL method in ultrasound presented high intra- and inter-rater reliability when measuring the coronal curvature in AIS subjects at the supine position, compared with the Cobb method in MRI.

### Validation of ultrasound assessment of spinal curvature in the coronal plane

To validate the ultrasound assessment of spinal curvature in the coronal plane, 3 main aspects were investigated: comparison of means, Bland-Altman limits of agreement and correlation coefficient (r) between ultrasound and MRI assessments in the patients with AIS. Table 3 lists the relevant statistical parameters of these 3 aspects. Furthermore, the impact of curve magnitude (Cobb angle degrees), variation in selected upper-end vertebra (UEV) and lower-end vertebra (LEV) between ultrasound and MRI, as well as the level of apical vertebra on the validity of ultrasound assessment of coronal curvature were investigated accordingly in the sample categories.

**Table 3:** Validation of coronal curvature assessments using ultrasound compared with MRI.

Variables	Curves,n	Bias	SD of bias	95% Limits of Agreement	Pearson Correlation Coefficient (*r*.)	T-test *Sig*. (2-tailed)
**Cobb angle degrees**						
Cobb angle: 10.2~68.2°	30	0.3°	1.4°	-2.4° ~ 3.1°	0.997	0.20
Cobb angle: 10.0~20.0°	19	0.0°	0.9°	-1.8° ~ 1.8°	0.934	0.93
Cobb angle: 20.0~45.0°	8	0.0°	1.1°	-2.2° ~ 2.2°	0.989	1.00
**Variation in selected UEV** ^**a**^						
variation = 0	10	0.4°	1.0°	-1.6° ~ 2.4°	0.998	0.23
variation = 1	14	0.6°	1.4°	-2.2° ~ 3.4°	0.998	0.15
variation = 2	6	-0.4°	1.8°	-3.9° ~ 3.1°	0.997	0.60
**Variation in selected LEV** ^**b**^						
variation = 0	13	0.3°	1.3°	-2.2° ~ 2.9°	0.997	0.37
variation = 1	11	0.5°	1.6°	-2.7° ~ 3.7°	0.998	0.33
variation = 2	6	0.0°	1.3°	-2.5° ~ 2.5°	0.989	0.98
**Level of apical vertebra**						
T1-T4	4	0.6°	0.8°	-1.0° ~ 2.1°	0.987	0.25
T5-T8	8	0.9°	1.9°	-2.9° ~ 4.6°	0.997	0.25
T9-T12	9	0.5°	0.8°	-1.2° ~ 2.1°	0.999	0.14
L1-L5	9	-0.4°	1.5°	-3.2° ~ 2.5°	0.997	0.50

UEV ^a^: upper-end vertebra; LEV ^b^: lower-end vertebra. The Bias, SD of bias and 95% Limits of Agreement are calculated from the Bland-Altman method.

### Validation: comparison of means between ultrasound and MRI

**Fig 4:**
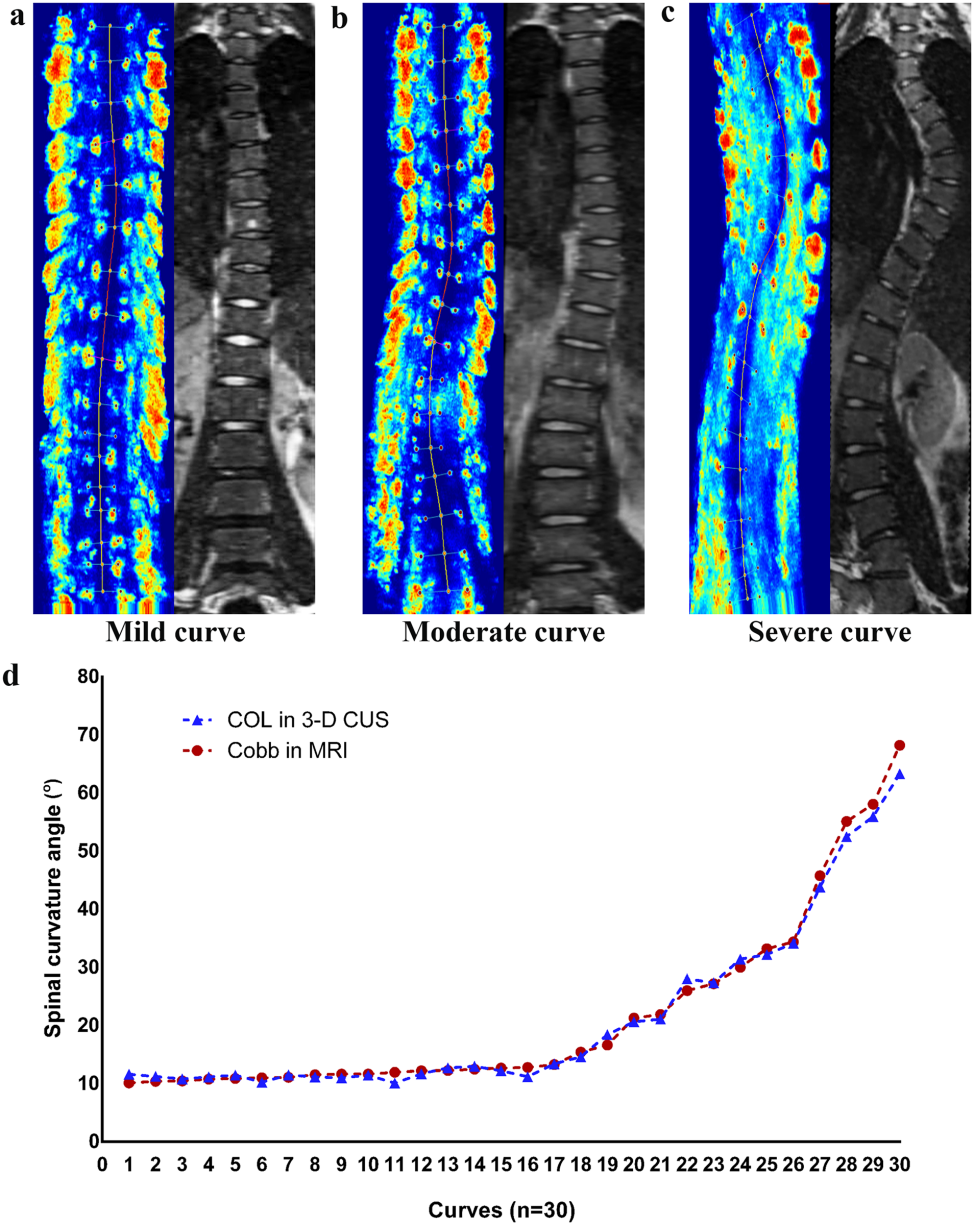
Comparison of ultrasound versus MRI images for AIS patients with. (a) Mild curve; (b) Moderate curve; (c) Severe curve; (d) A scatter plot of the COL method in ultrasound versus the Cobb method in MRI for the entire curve cohort.

For the entire curve cohort (n = 30), the mean value of coronal curvature angle measured by the COL method in ultrasound was 21.3°±15.9° while the average value by the Cobb method in MRI was 21.7°±15.1°. As shown in Fig 4, the curve profiles presented in the coronal plane of ultrasound images were similar to that of MRI images in AIS patients with mild, moderate and severe curvature angles (Fig 4a–4c); the two dashed lines in the scatter plot representing the angle measured by the COL method in ultrasound versus the Cobb method in MRI respectively were almost identical when measuring coronal curvature in the entire curve cohort (Fig 4d). Moreover, the Paired *t*-test results showed that there was no significant difference between these two methods (Table 2).

### Validation: Bland-Altman method

**Fig 5:**
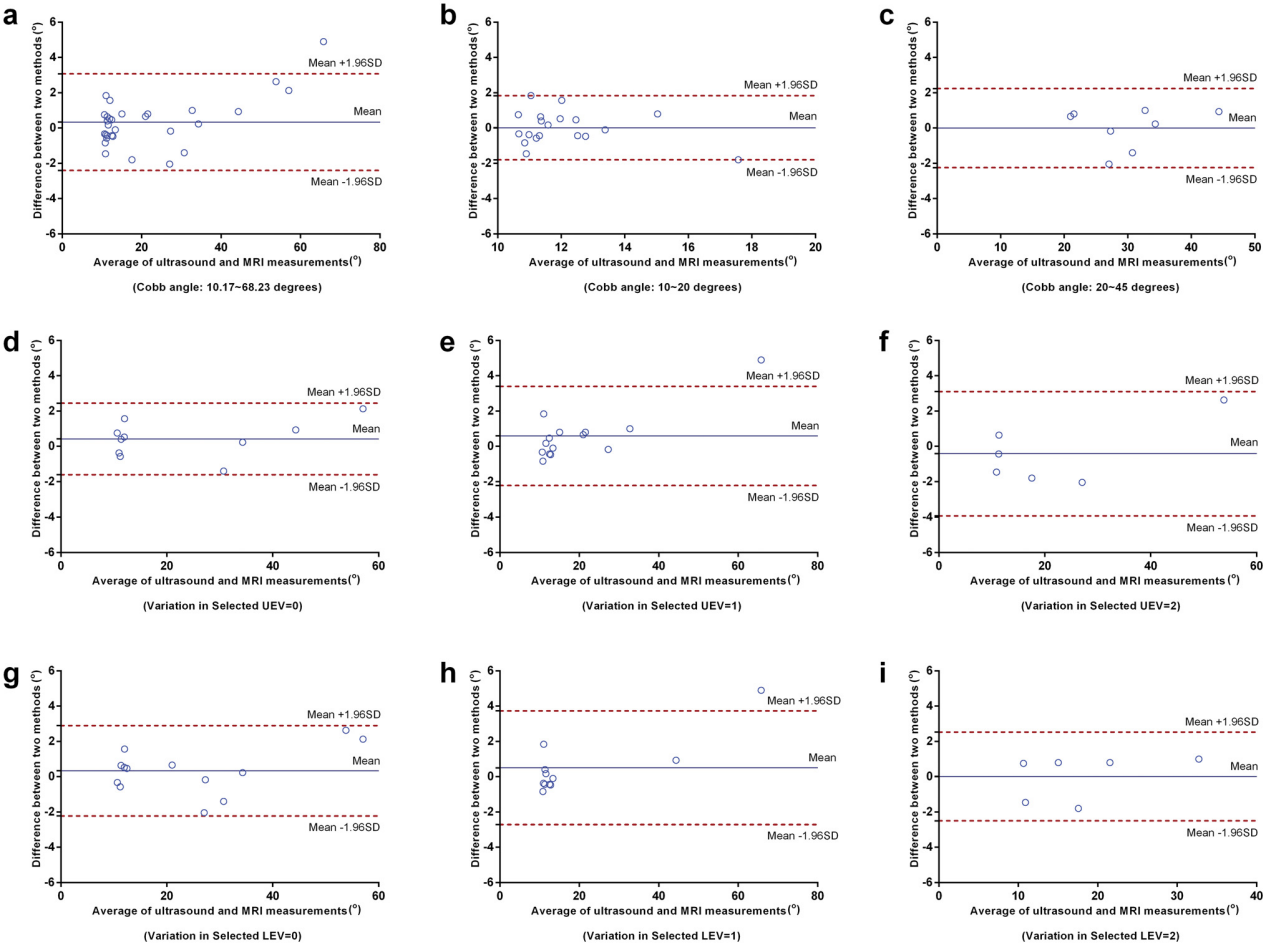
Bland–Altman plot assessing the agreement of coronal curvature measurements using ultrasound and MRI in the sample categories. (a) Cobb angle: 10.2°~68.2°; (b) Cobb angle: 10.0°~20.0°; (c) Cobb angle: 20.0°~45.0°; (d) Variation in selected UEV = 0; (e) Variation in selected UEV = 1; (f) Variation in selected UEV = 2; (g) Variation in selected LEV = 0; (h) Variation in selected LEV = 1; (i) Variation in selected LEV = 2. The central line represents mean differences (Bias); upper line shows mean+1.96SD and lower line mean-1.96SD. UEV: upper-end vertebra; LEV: lower-end vertebra.

**Fig 6:**
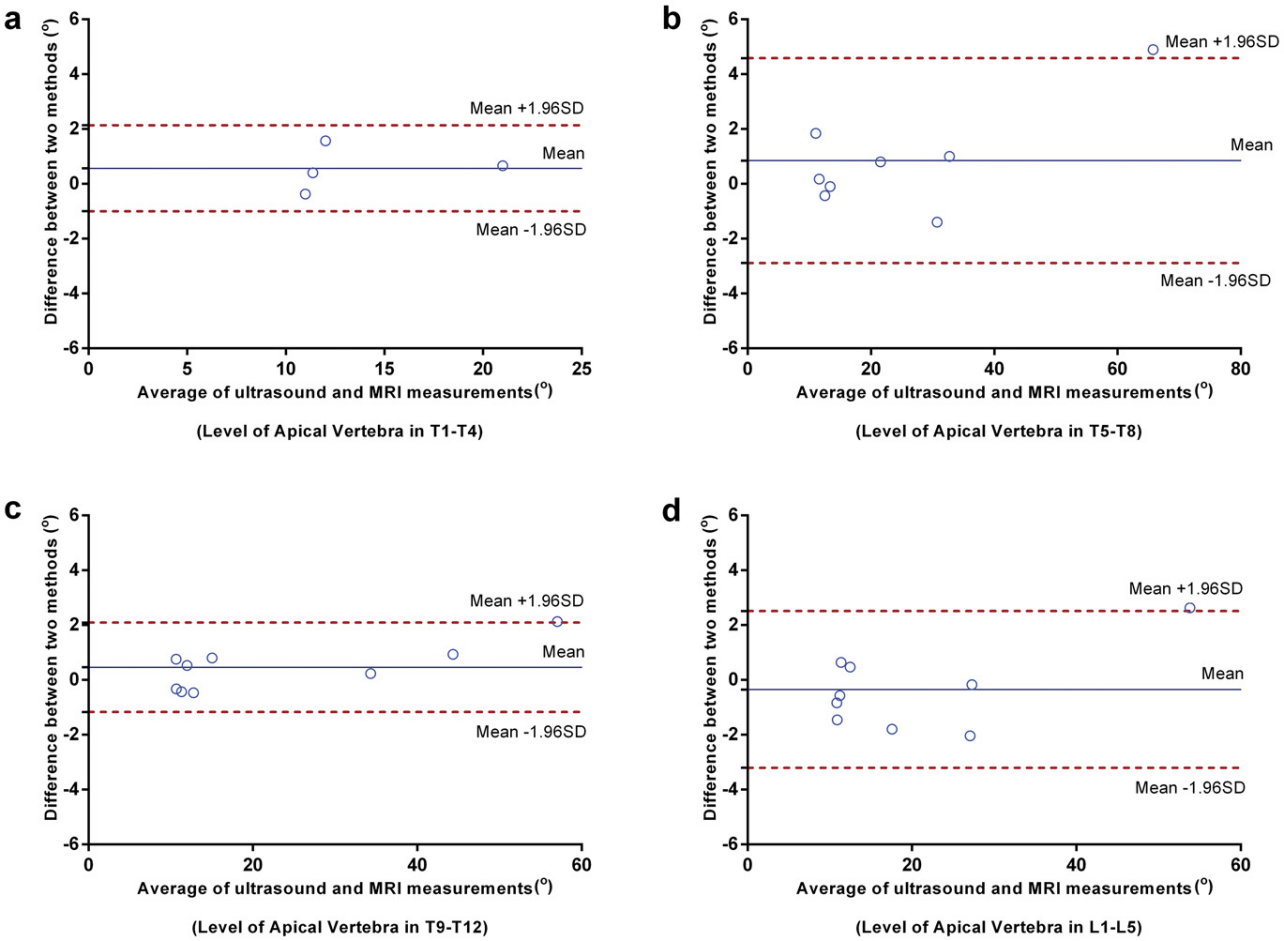
Bland–Altman plot assessing the agreement of coronal curvature measurements using ultrasound and MRI in the sample categories. (a) Level of apical vertebra in T1-T4; (b) Level of apical vertebra in T5-T8; (c) Level of apical vertebra in T9-T12; (d) Level of apical vertebra in L1-L5.

The agreement between the COL method in ultrasound and the Cobb method in MRI was demonstrated using the Bland–Altman method, which consisted of a scatter plot of the two measurements difference against the average of the two measurements (Figs 5 and 6), as well as bias and limits of agreement calculated (Table 3). This method is the most popular statistical method for assessing agreement between two methods of clinical measurements [28]. As shown in Fig 5, the Bland-Altman plot revealed good agreement between ultrasound and MRI measurements of coronal curvature in the overall cohort (Fig 5a), as well as in the samples with Cobb angle of 10.0°~20.0° (Fig 5b) and in those with Cobb angle of 20.0°~45.0° (Fig 5c). Almost all the measurements clustered around the central lines except the outliers with Cobb angle larger than 60.0°, showing low discrepancy between mean difference and limits of agreement.

Notably, the samples with variation in selected UEV/LEV (equal to 0) showed lower discrepancy with respect to mean difference than the others with variation in selected UEV/LEV (equal to 1 or 2) (Fig 5d–5i). This indicated that the variation in selected end vertebra between ultrasound and MRI may decrease the agreement between these two methods. In addition, the samples with level of apical vertebra in T5-T8 presented larger discrepancy than the others. However, this may be due to the influence of outlier with Cobb angle larger than 60.0° in this plot (Fig 6).

The corresponding values of Bland-Altman bias, SD of bias and 95% limits of agreement were provided in Table 3. The bias between coronal curvature measured using ultrasound and MRI ranged from 0.0° to 0.9°. For the entire cohort, the 95% limits of agreement were -2.4° ~3.1°, the absolute difference (5.5°) of which was slightly larger than the commonly accepted difference (5°) between successive curvature measurements [29, 30]. This suggested that the agreement between ultrasound and MRI measurements of coronal curvature was almost within the accepted threshold of curvature measurement. In addition, the largest 95% limits of agreement were -2.9°~4.6° for the sample with the level of apical vertebra in T5-T8, while the least were 1.0°~2.1° for the sample with the level of apical vertebra in T1-T4.

### Validation: Pearson correlation analysis

**Fig 7:**
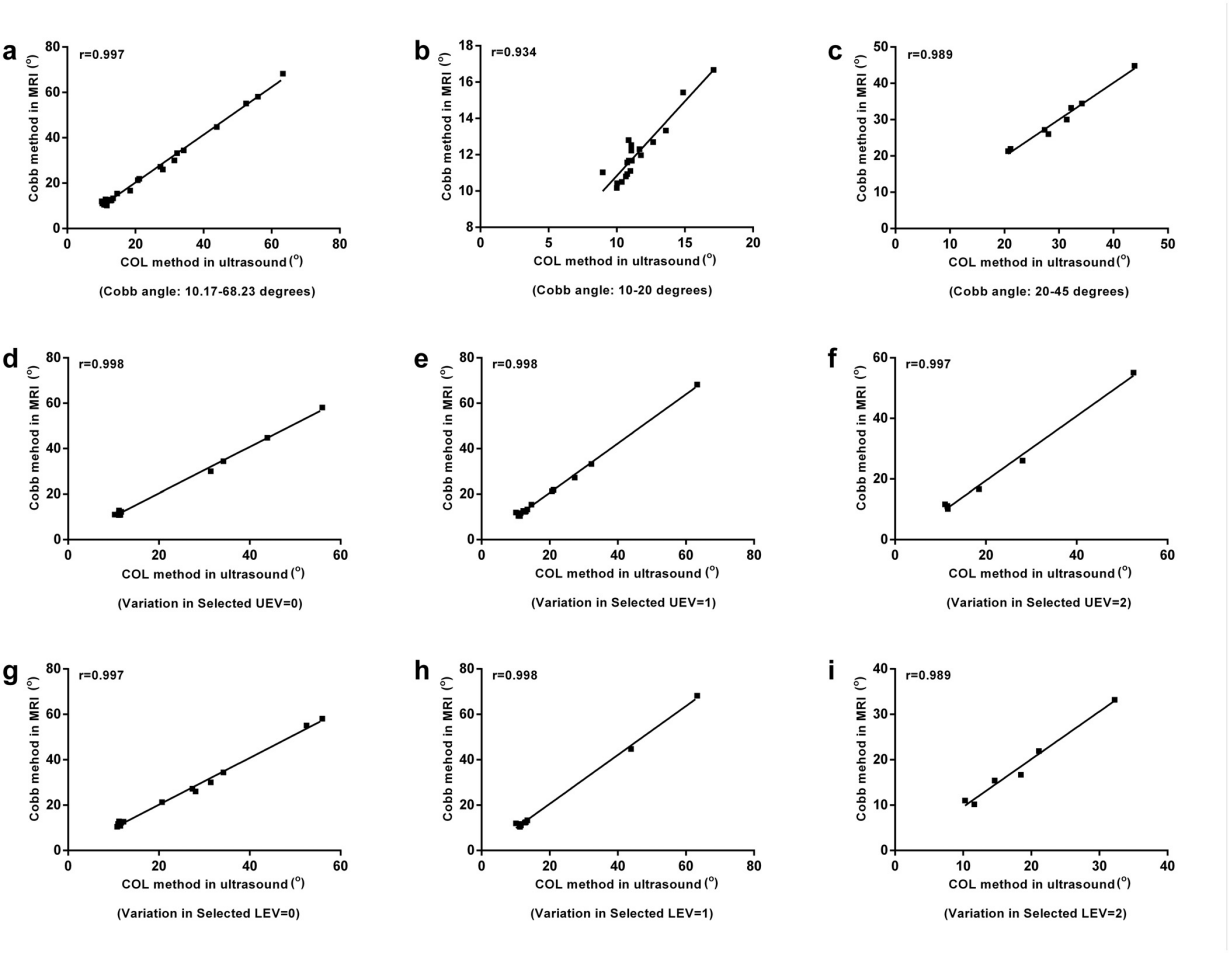
Correlation of coronal curvature measurements using ultrasound and MRI in the sample categories. (a) Cobb angle: 10.2°~68.2°; (b) Cobb angle: 10.0°~20.0°; (c) Cobb angle: 20.0°~45.0°; (d) Variation in selected UEV = 0; (e) Variation in selected UEV = 1; (f) Variation in selected UEV = 2; (g) Variation in selected LEV = 0; (h) Variation in selected LEV = 1; (i) Variation in selected LEV = 2. UEV: upper-end vertebra; LEV: lower-end vertebra.

**Fig 8:**
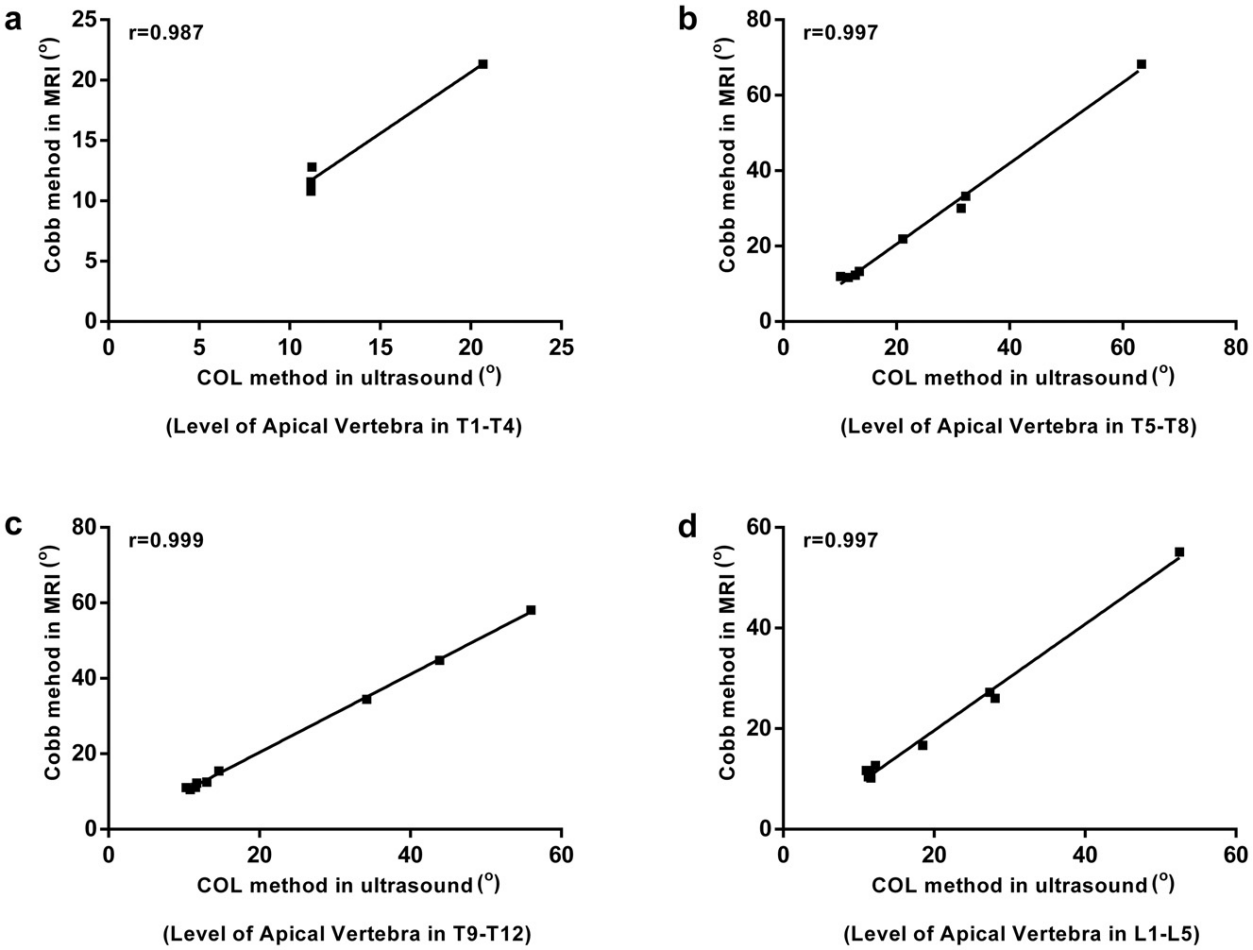
Correlation of coronal curvature measurements using ultrasound and MRI in the sample categories. (a) Level of apical vertebra in T1-T4; (b) Level of apical vertebra in T5-T8; (c) Level of apical vertebra in T9-T12; (d) Level of apical vertebra in L1-L5.

The Pearson correlation analysis showed the similar trend observed using Bland-Altman method (Figs 7 and 8). The correlation between ultrasound and MRI assessments of coronal curvature was found to be high for all the sample categories (correlation coefficient r>0.9, P < 0.05) (Table 3).

Taken together, the validity of the ultrasound assessment of spinal curvature in the coronal plane was demonstrated by comparison with MRI measurement at the supine position.

## Discussion

In the present study, the reliability and validity of ultrasound assessment of spinal curvature in the coronal plane were investigated respectively. The major findings of this study are: (1) The COL method in ultrasound showed high intra- and inter-rater reliabilities to measure the coronal curvature; (2) Compared with the Cobb method in MRI, the COL method in ultrasound showed no significant difference when measuring coronal curvature at supine position; (3) Bland-Altman method demonstrated a strong agreement between ultrasound and MRI assessments in quantifying the spinal curvature in the coronal plane; (4) The high correlation between ultrasound and MRI measurements of coronal curvature was found in all the sample categories.

Currently, the application of ultrasound has been studied in the assessment of the coronal curvature in patients with AIS. The COL method has been proposed by Lou and his colleague to approximate the Cobb angle from ultrasound image. The intra- and inter-rater ICC (2,1) values of the COL method in ultrasound were reported to be above 0.80; the SEM less than 2.8° [15, 23, 24]. In the present study, the results were consistent with the previous studies. The intra- and inter-rater ICC (2,k) values of ultrasound assessment were greater than 0.9; the intra- and inter-rater MAD, SD and SEM were less than 2.1°, 2.7°, and 2.2° respectively. Moreover, the intra- and inter-rater reliability of the COL method in ultrasound was similar to that of the Cobb method in MRI. Besides, the reliability results of ultrasound measurement in this study were comparable to the intra- and inter-rater statistics of radiographic measurement reported in the literature: MADs ranged from 1.2° to 7.0° [4–6], and ICCs ranged from 0.88 to 0.99 [6–8]. This indicated that the reliable measurements can be obtained by the COL method in ultrasound when measuring the coronal curvature for patients with AIS.

On the basis of reliability results, the validity of the COL method in ultrasound was investigated. In previous studies, Lou et al. reported that the difference between spinal curvature angles measured using the COL method in ultrasound and the Cobb method in radiograph ranged from 0.2° to 1.4°; the correlation between these two methods was high for mild and moderate AIS patients. This study showed comparable results to the previous studies when MRI was chosen to be the reference. MRI can provide a clear 3-D image of the scoliotic spine without radiation exposure [31], which is similar to ultrasound. Our study demonstrated that the coronal curvature measured by ultrasound showed no significant difference but strong correlation with MRI at the supine position.

In addition, the validity of ultrasound assessment was investigated by the Bland-Altman method, which is the most popular statistical method for assessing agreement between two methods of clinical measurements [28]. In this method, the 95% limits of agreement are the estimates of values, which mean 95% of differences between two methods will lie between these limits [26]. The larger 95% limits of agreement are, the more discrepancy between the two methods will be. Currently, the variation within 5.0° between successive curvature measurements has been considered to be acceptable clinical error [29, 30]. Therefore, it is postulated that the accuracy of the COL method of ultrasound will be validated if the 95% limits of agreement are within 5.0° between ultrasound and MRI assessments. In the present study, 95% limits of agreement were reasonably narrow not extending over the acceptable clinical error (5.0°) in most cases. These results demonstrated the validity of the COL method in ultrasound when measuring spinal curvature in the coronal plane. However, the variation in selected UEV/LEV (equal to 1 or 2) and level of apical vertebra (T5-T8) enlarged the 95% limits of agreement (> 5.0°). It appears that the end-vertebra selection error and different apical vertebral level may influence the agreement between the COL method and Cobb method and lead to inaccurate measurement of coronal curvature using ultrasound. The possible reasons for these maybe the lower resolution of ultrasound images, lack of experience, as well as the changed posture of subjects between ultrasound and MRI scans. Therefore, future studies are required to investigate how to improve the ultrasound images in order to reduce the end-vertebra selection error.

It is noteworthy that some curves or landmarks were missing in some ultrasound images. This resulted in difficult identification of landmarks in ultrasound image and inaccurate measurement. There are several possible reasons for the missing information in ultrasound images. First, the bad contact between transducer and subjects' back, especially for a large rib hump, would result in loss of ultrasound information. In order to ensure a good surface contact, Li et al. designed a silicon sleeve attached to the ultrasound transducer [11]. Therefore, much attention should be paid on how to create a good surface contact between transducer and subjects' back for ultrasound scanning in future studies. Second, the thick muscles, in particular of the lumbar region, would cause ultrasound signal penetration reduced and lower resolution of ultrasound images. Third, the vertebral rotation would make it difficult to cover all the information during ultrasound scanning.

Although the results of this study have demonstrated the validity and reliability of COL method in ultrasound, there are still some limitations. The eligible curves in this study involved a whole range of curve severity of AIS patients. However, the severe curves accounted for a small proportion in all the analyzed curves. Therefore, further research is still needed to validate the proposed ultrasound assessment in a larger sample size. Besides, the semi-automatic program applied in reconstruction of 3-D ultrasound images, identification procedure of landmarks and data measurement took around 5 minutes for one trial of ultrasound measurement. Thus, it is necessary to develop an automatic program to facilitate the ultrasound measurements. As well, the ultrasound scanning skills at the supine position should be also improved for accurate measurements of scoliotic spines.

## Conclusion

The radiation-free ultrasound assessment appeared to be a reliable & valid method for measuring spinal curvature in the coronal plane. The COL method in ultrasound has been validated in comparison with the Cobb method in MRI. Continuous studies are required to optimize the ultrasound scanning and measuring procedure, and to further validate the ultrasound measurements in the other anatomical planes. With these efforts, ultrasound will become a potential option used as an alternative to radiography for screening and routine assessment of scoliosis and other spinal deformities.
